# Effect of Women's Decision-Making Autonomy on Infant's Birth Weight in Rural Bangladesh

**DOI:** 10.1155/2013/159542

**Published:** 2013-12-12

**Authors:** Arpana Sharma, Manzur Kader

**Affiliations:** ^1^Department of Community Medicine, Manipal College of Medical Sciences (MCOMS), 155 Pokhara, Nepal; ^2^Department of Women's and Children's Health, International Maternal and Child Health (IMCH), Uppsala University, 751 85 Uppsala, Sweden

## Abstract

*Background*. Low birth weight (LBW), an outcome of maternal undernutrition, is a major public health concern in Bangladesh where the problem is most prominent. Women's decision-making autonomy is likely an important factor influencing maternal and child health outcomes. The aim of the study was to assess the effect of women's decision-making autonomy on infant's birth weight (BW). *Methods*. The study included data of 2175 enrolled women (14–45 years of age) from the Maternal and Infant Nutritional Intervention in Matlab (MINIMat-study) in Bangladesh. Pearson's chi-square test, analysis of covariance (ANCOVA), and logistic regression analysis were applied at the collected data. *Results*. Women with lowest decision-making autonomy were significantly more likely to have a low birth weight (LBW) child, after controlling for maternal age, education (woman's and her husband's), socioeconomic status (SES) (odds ratio (OR) = 1.4; 95% confidence interval (CI) 1.0, 1.8). BW was decreased significantly among women with lowest decision making autonomy after adjusting for all confounders. *Conclusion*. Women's decision-making autonomy has an independent effect on BW and LBW outcome. In addition, there is a need for further exploration to identify sociocultural attributes and gender related determinants of women decision-making autonomy in this study setting.

## 1. Background 

WHO has defined low birth weight (LBW) as birth weight (BW) less than 2500 g at birth which is a global public health concern [1]. About one half of the world's low birth weight (LBW) babies are born in South Asia and Bangladesh has the highest incidence (31–47%) [1, 2]. BW is an important predictor of infant growth and survival and is strongly associated with early mortality and morbidity with adverse long-term outcomes [3, 4].

Low weight at birth is either the result of preterm delivery or intrauterine growth retardation (IUGR) [5]. BW is affected by various factors including maternal age, parity, BMI, quality of antenatal care, anaemia, and pregnancy induced hypertension (PIH) [6–10]. However, in developing countries like Bangladesh maternal undernutrition is a major determinant of LBW [11–15].

It has been reported that in many South Asian countries including Bangladesh women's socioeconomic status is low and gender inequality persists in many sectors starting from intrahousehold food allocation, education, work, and property rights to decision-making matters. The majority of women have limited access to and control over resources and restriction in their mobility and are often under threat of violence from male relatives [16–18].

Women's autonomy is a multidimensional concept that remains ill-defined. There is no single accepted definition that represents it well. In this study, women's decision-making autonomy is defined as women personal power in the household and her ability to make and execute independent decisions of her own concern or about close family members which is closely associated with maternal and child health outcomes [19–21].

In a recent study in South India, it was observed that women with higher decision-making autonomy on financial resources and freedom to go to the market were significantly less likely to have a stunted child, after controlling for socioeconomic status and mother's education [20]. There have been a number of other studies that examine different dimension of women's autonomy and its relationship with reproductive health and health outcomes such as maternal and child health care utilisation [22, 23], reproductive preferences [24], and contraceptive use [25, 26]. A study done by Bloom et al., 2001, in North India shows that women's autonomy is the major determinant of maternal health care utilisation. Women with greater freedom of movement are more likely to receive antenatal care and to use delivery care, and the authors suggested that women's autonomy is equally important to educational and economic characteristics.

In order to address the LBW issue successfully, in a country like Bangladesh, where maternal undernutrition and LBW are prevalent, it could be important to explore the role of women's decision-making autonomy in relation with birth weight. The aim of this study is to assess the effect of women's decision-making autonomy on infant's birth weight in rural Bangladesh. We hypothesized that women with lowest decision-making autonomy are more likely to have LBW babies than women with highest decision-making autonomy.

## 2. Methods

### 2.1. Study Setting

The study was conducted in Matlab, a poor rural subdistrict within Chandpur district in Bangladesh, located about 55 km southeast of Dhaka, the capital. The main economic activities of the area are farming and fishing. Rice and fish are the common staple food.

The women status is low in general, and traditional social and cultural norms curtail the women's autonomy. It has been reported that, although in recent years the freedom and status of women have improved in this setting due to various women empowerment programs such as microcredit organization and other NGOs, it is still far from advantageous. It is unusual for the majority of women to leave their home without a male companion which limits their opportunities for employment outside their homestead [27]. A girl usually marries in her teens and moves into her husband's home and loses the support network from her native family [17].

Since 1966 International Centre for Diarrhoeal Disease Research, Bangladesh (ICDDR, B), has run a Health and Demographic Surveillance System (HDSS) in Matlab covering a population of 220,000 that has collected vital demographic information (births, deaths, fertility, migrations, marriages and divorces, and household divisions). These data are updated by monthly home visits by trained Community Health Research Workers (CHRW) [27].

### 2.2. Study Subjects

The study included data from the Maternal and Infant Nutritional Intervention in Matlab (MINIMat study), which is a combined intervention trial to promote maternal and infant health by providing prenatal food and micronutrients supplementation during pregnancy. Within HDSS system, pregnant women were identified by trained CHRWs on their monthly household visits. All pregnant women aged 14–45 years in the area were eligible for enrolment in the study. The inclusion criteria were gestation less than 14 weeks with ability to give consent. Urine pregnancy tests were performed in the field whenever a woman reported that her last menstrual period (LMP) was overdue by two weeks or more or that she understood that she was pregnant and later confirmed by ultrasonography at her first clinic visit. After confirmation of pregnancy women were closely followed throughout their pregnancy until delivery. Out of 4436 enrolled, 2175 participants who had complete information on birth weight and decision-making autonomy were included in the analysis.

### 2.3. Data Collection and Quality Control of Data

ICDDR, B was responsible for all data collection. Data were collected by trained interviewers and paramedics from November 2001 to October 2003. Structured questionnaires with precoded questions were used for data collection. All questionnaires were pretested and revised accordingly by the responsible investigators before introducing them in the study.

A female field assistant measured weight and height of all enrolled women and collected sociodemographic information including women's age, parity, educational status, income, household assets, husband's education, and decision autonomy. We used the wealth index created by MINIMat team from the information on household assets to measure socioeconomic status (SES) of women [28]. All collected data were routinely reviewed once every two weeks by the responsible investigators.

### 2.4. Ethical Considerations

Written consent was obtained from each woman before enrolment in the study. The women were informed that they had the right to withdraw from the study at any point without any consequences for access to or use of routine ICDDR, B and government health care services. Confidentiality of information was strictly maintained. Ethical approval for this study was obtained from the Ethical Review Committee of ICDDR, B.

### 2.5. Anthropometric Measurements

#### 2.5.1. Birth Weight

Research assistants were trained to carry out the anthropometric measurements as per WHO guidelines. Weighing equipment was standardized daily with standard weights. Women were provided with a “birth notification” card that should be sent to the study office immediately after delivery. It also contained an incentive to do so by promising a small amount of money to reimburse travel cost. The infant birth weight was measured within 72 hours of delivery by using beam scales (Seca GmbH), with a precision of 10 g. We used WHO cutoff for low birth weight (LBW) which refers infants born weighing less than 2.5 kg, regardless of gestational age and the cause of LBW.

#### 2.5.2. Maternal Body Mass Index (BMI)

The weight of the pregnant women was measured with a precision of 0.1 kg with electronic scales (UNISCALE) that were accurate to 100 g. Height was measured to a precision of 0.1 cm by using a stadiometer. BMI was calculated as wt (kg)/ht. (m)^2^. We followed the WHO definition of BMI (i.e., <18.5 for mild undernutrition) as the cutoff for low BMI and high BMI was >24.5.

#### 2.5.3. Decision-Making Autonomy

Decision-making autonomy was estimated from 6 questions on decision making, for example, decision regarding own health care, making small and large household purchases. These questions were originally developed and validated from Bangladesh Demographic and Health Survey BDHS [29]. In order to obtain information on the above measures of women's decision-making autonomy, each woman enrolled in the study was asked the following questions at the time of her first antenatal visits: “To what extent are you able to influence decisions related to the following”:

- When you are sick, your own health care?
- Making household purchases for household needs?
- Making large household purchases?
- Visits to natal relatives?
- Consumption of food that you like to eat?
- Utilisation of contraceptive methods?

To develop a score for analysis, the responses were coded as follows: (a) 3 points for decisions made by the women; (b) 2 points for women who have some influence on decision-making; and (c) 1 point for those who do not have any influence or a very little influence.

To facilitate analysis, a composite score ranges from 6 to 18 with a mean score 12.9 was created to measure decision making autonomy, which was further divided into tertile categories, resulting in the final score with lowest autonomy (≤12), medium (13-14), and highest autonomy (≥15).

### 2.6. Statistical Analysis

Differences in enrolment characteristics between the women with and without complete data on birth weight (BW) and decision-making autonomy were examined by using independent *t*-test and chi-square tests based on the type of variables (continuous/categorical). Bivariate association of BW with each covariate was assessed using the analysis of variance (ANOVA), independent *t*-test and chi-square tests. Covariates considered in the analysis comprised maternal age, parity, BMI, education of woman and her husband, household income, asset score, and living with mother-in-law. Confounding factors were included in the final analysis for adjustment if its influence on the effect estimate was found to be more than 10%. Finally, ANCOVA and binary logistic regression were conducted with BW as a continuous and categorical dependent variable for both crude (model with decision-making autonomy only) and adjusted models (full model adjusted with confounding factors). All the results are reported as Mean ± SD unless stated otherwise. A *P* value less than 0.05 was considered as the level of statistical significance. All statistical analyses were carried out using IBM SPSS Statistics 19 for Window (SPSS Inc., Chicago, IL).

## 3. Results

**Figure 1:**
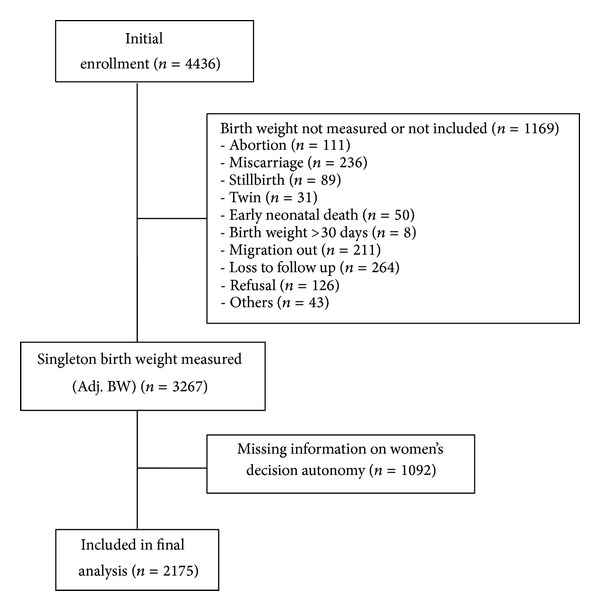
Study participation.

Out of 4436 women in the study group, 2175 (*≈*49%) with complete information on BW and decision-making autonomy were included in the final analysis. In the first step about 26% of the participants were excluded for missing information on BW while 24% were excluded later for missing information on decision-making autonomy (Figure 1). Women with complete data on BW and decision-making autonomy were slightly older and had more children, lower education, and less income. There were no significant differences in maternal height, BMI, husband education, and residing with mother-in-law between those with or without complete data. Overall women in complete group had higher mean BW and lower percentage of LBW infants than in incomplete group (Table 1).

### 3.1. Decision-Making Autonomy

Women in this study group appeared to hold very little decision-making power in large household purchases whereas majority (74%) of women had their final say over food like to eat. About half of the women had some influence on decision making regarding their own health care but very few of women had final say on it. Similarly, very few women had final say on using contraception, visits to natal relatives, and making small household purchases for daily household needs (Table 2).

### 3.2. Effect of Decision-Making Autonomy on BW

The mean BW of children born to women with highest decision-making autonomy was significantly higher (2.75 ± 0.4 kg) than in women with lowest decision-making autonomy (2.67 ± 0.3 kg). In multivariate analysis, BW was significantly lower (*≈*69 g) in women with lowest decision-making autonomy as compared to women with highest decision-making autonomy after controlling for confounding factors like maternal age, BMI, asset scores, women and her husband's education. Maternal age, BMI, a woman education and asset scores were also found to have significant effect on birth weight (Table 3).

The proportion of LBW infants was significantly lower (24%) in women with highest decision-making autonomy than in women with lowest decision-making autonomy (32%). Women with lowest decision-making autonomy had 40% increased risk of having LBW babies as compared to women with highest decision-making autonomy (odds ratio (OR) = 1.4; 95% confidence Interval (CI) 1.0, 1.8). Among other covariates, maternal age and asset scores remained significantly associated with LBW in the final model (Table 4) (results were shown already).

## 4. Discussion

The findings of this study support the hypothesis that low maternal decision-making autonomy is associated with increased LBW outcome in rural Bangladesh. Women with lowest decision-making autonomy had lighter babies as compared to women with highest decision-making autonomy.

There are many direct and indirect complex pathways underlie in the relationship between women decision-making autonomy and infant's BW. Women decision-making autonomy can affect infant BW by affecting women's health and nutritional status and foetal growth.

Women's low social status in Bangladesh followed by lack of decision-making autonomy on cooking or food choices can influence her health and nutritional status directly through dietary discriminatory practices such as unfair food sharing, inadequate and improper diet intake during pregnancy giving rise to maternal undernutrition and LBW outcome [12, 30].

Maternal under nutrition is a major determinant of LBW in a developing country like Bangladesh. The proportion of babies born with LBW reflects poor maternal health and nutritional status not only during pregnancy but over the whole life cycle of their childhood and young lives [3]. Poor maternal nutritional status at conception, low gestational weight gain due to inadequate dietary intake, and short maternal stature due to mother's own childhood malnutrition and micronutrients deficiencies are responsible for maternal under nutrition [12, 13]. Based on this concept, we can hypothesize that women with low decision-making autonomy on cooking or food choices are more likely to have LBW infants due to under nutrition and impaired foetal growth in-utero.

Woman's autonomy regarding decision making on her own health care is also closely linked with maternal and child health outcome. A study based in three South Asian countries revealed that decisions of women's health care were made without their participation for the majority of women in Nepal, approximately half in Bangladesh (54.3%) and (48.5%) in Indian households [21]. This finding is consistent with our findings which reflects that only 29% of women had a final say on decision making of their own health care and 67% of them had only some influence. Women with lack of decision-making autonomy on their health care are less likely to obtain regular health checkups including antenatal care, which covers iron and folic acid supplementation, Tetanus toxoid immunization, safe delivery practices, and important health information regarding pregnancy and childbirth. Beside this, low level of decision-making autonomy on health care can lead to low uptake of prenatal food and micronutrient supplementation in our study setting. Lack of this could contribute to poor prenatal care, poor maternal health and nutritional status, and impaired foetal growth leading to LBW.

A study in South India suggested that women's permission to go to the market or to visit natal relatives could potentially provide a forum for exchanging health related information and could receive prenatal care earlier in their pregnancy and treatment for disease associated with IUGR such as hypertension and heart disease that may lead to better birth outcome [20]. On the other hand, other studies have explored the relationship between women autonomy and maternal health care utilisation using different aspects of autonomy such as financial autonomy, decision-making autonomy, and mobility autonomy. The results revealed that women with greater freedom of movement obtained better antenatal care and were more likely to use safe delivery care which can affect birth weight [19, 22].

Apart from this study, many other studies have examined the role of women's autonomy in fertility preferences and use of contraception [24, 26]. Women's final say in decision regarding day to day household purchases and spousal communication is significantly associated with fertility preferences and use of contraception which may facilitate proper birth spacing and prevent early pregnancy and childbirth which can also help to reduce the LBW prevalence [25].

Women in South Asian countries including Bangladesh are often under threat of violence due to their subordinate position [16]. They may have less possibility for decision making in the household in different sectors such as decision making for herself or her family members, purchasing food, control over resources, and so forth, which might affect her health and nutritional status as well as foetal growth and development, giving rise to LBW babies [31].

A study in Bangladesh reported that 50% of women experiencing some form of family violence had LBW babies followed by early childhood growth impairment. We hypothesize that women with low decision-making autonomy are more likely to face any kind of violence which might affect infant's birth weight through several mechanisms. Direct physical trauma to pregnant women's abdomen may lead to preterm delivery along with other serious maternal and foetal complications. It has been reported that stress and depression related with all forms of violence can increase the risk of impaired foetal growth and LBW [31]. Antenatal depression was also found to be associated with foetal growth retardation [32].

Many researchers have suggested that stress and depression cause disturbances in hypothalamic-pituitary-adrenal axis giving rise to increased level of stress hormones which affects intrauterine environment resulting in IUGR. Similarly, activation of sympathetic-adrenal-medullary system resulting in vasoconstriction and hypoxia with decreased uteroplacental perfusion contributes to growth restriction and LBW. However, further exploration is required to find out the exact mechanism [31, 33]. Along with stress and depression, violence is often accompanied by social isolation and lack of social support which may affect infant's BW [33].

A major strength of the study is randomized enrolment of pregnant women over the survey period which makes the results relatively representative of the study population, and hence the findings can be appropriately generalized to women in similar rural areas of Bangladesh. The socioeconomic characteristics of this study rural area in Matlab were similar to those of national level according to the census of Matlab HDSS study area in 2005. These findings in Matlab can be applied to or are important to other populations in the world.

However, there are some limitations and some sources of bias in this study.

Understanding the role of women's decision-making autonomy in relation to BW is complex because of its multidimensionality and difficulty in formulating an appropriate autonomy measures. In this study, we have examined only one aspect of women's autonomy, that is, decision-making autonomy. Different dimensions of autonomy might have different influence on infants BW. Along with decision-making autonomy we could have analysed other areas of autonomy like control over finances, freedom of movement, and woman's attitude towards domestic violence (wife beating) which could have important impact on BW in this context.

Women in our study group were significantly older and had more children which is likely to overestimate the effect. We have adjusted BMI as a confounding factor in the final multivariate model with BW as a continuous variable which might cause overadjustment and underestimate the effect.

## 5. Conclusion

This study revealed that women's decision-making autonomy has an independent effect on infant BW and LBW outcome after controlling for all confounders like maternal age, SES, and education. The result of our study suggests that improving women's decision-making autonomy will have a positive effect on reduction of LBW. In addition, there is a need for further exploration to identify sociocultural attributes and gender related determinants of women's decision-making autonomy in this study setting.

## Figures and Tables

**Table 1:** Baseline characteristics of the participants with complete and incomplete information.

Characteristic	Complete data *N* = 2175	Incomplete data *N* = 2261	*P* value^†^
Age (years)			
Mean age (SD)	26 ± 5.89	25.60 ± 6.05	**0.028**
≤18	195 (9.0)	253 (11.3)	**0.040**
19–34	1776 (81.7)	1784 (79.4)	
≥35	204 (9.4)	210 (9.3)	

Parity			
0	681 (31.3)	819 (36.4)	**0.005**
1-2	1087 (50)	1034 (46.0)	
3-4	335 (15.4)	324 (14.4)	
≥5	70 (3.2)	71 (3.2)	

Height (cm)			
Mean ht. (SD)	149.74 ± 5.34	149.79 ± 5.31	0.732

Weight (kg)			
Mean wt. (SD)	45.28 ± 6.74	45.47 ± 7.08	0.361

BMI (kg/m^2^)			
≤18.49	596 (27.4)	620 (27.5)	0.757
18.50–24.99	1454 (66.9)	1493 (66.3)	
≥25	123 (5.7)	139 (6.2)	
Mean (SD)	20.17 ± 2.64	20.23 ± 2.73	0.464

Women education (y)			
0	674 (31.0)	755 (33.4)	**0.025**
1–5	500 (23.0)	448 (19.8)	
>5	1001 (46.0)	1058 (46.8)	
Mean (SD)	5 ± 4.04	5.04 ± 4.23	0.800

Lives with mother-in law			
No	956 (44.1)	937 (41.6)	0.085
Yes	1210 (55.9)	1317 (58.4)	

Husband education (y)			
0	670 (31.0)	676 (30.1)	0.655
1–5	510 (23.6)	519 (23.1)	
>5	982 (45.4)	1051 (46.8)	
Mean (SD)	5.37 ± 4.54	5.54 ± 4.65	0.239

A stable household of income			
No	1247 (57.3)	1183 (52.3)	**0.001**
Yes	928 (42.7)	1078 (47.7)	

Household income and expenditure situation in last year			
Surplus	564 (26.0)	635 (28.2)	0.205
Expenditure equaled income	1171 (54.0)	1200 (53.2)	
Occasional deficit	376 (17.3)	351 (15.6)	
Constant deficit	58 (2.7)	68 (3.0)	

Calculated asset scores			
Poor	414 (19.0)	474 (21.0)	0.404
Below middle	454 (20.9)	437 (19.3)	
Middle	425 (19.5)	458 (20.3)	
Upper middle	442 (20.3)	445 (19.7)	
Rich	440 (20.2)	447 (19.8)	

Birth weight (g)			
<2500	646 (29.7)	351 (32.1)	0.153
≥2500	1529 (70.3)	741 (67.9)	
Mean (SD)	2704.72 ± 407.90	2672.33 ± 415.01	**0.033**

^†^Differences assessed with independent *t*-test for continuous variables and with Pearson's chi-square tests for categorical variables. Bold font refers to statistical significance.

**Table 2:** Summary statistics of decision autonomy scores (*n* = 2175).

Characteristics	(1) A little or no influence *N* (%)	(2) Some influence *N* (%)	(3) A lot or sole decision making *N* (%)
(1) Decision regarding own health care, when fall sick	78 (3.6)	1465 (67.4)	632 (29.1)
(2) Making small household purchases for household needs	393 (18.1)	1039 (47.8)	743 (34.2)
(3) Making large household purchases	943 (43.4)	1149 (52.8)	83 (3.8)
(4) Visit to natal relatives	292 (13.4)	1238 (56.9)	645 (29.7)
(5) Consumption of foods like to eat	80 (3.7)	486 (22.3)	1609 (74.0)
(6) Use of contraception	173 (8.0)	1659 (76.3)	343 (15.8)

**Table 3:** Association of BW with decision-making autonomy using analysis of covariance (ANCOVA) with crude and adjusted models; *B* (coefficient) and 95% confidence intervals (CI).

	Model with autonomy only		Full model^1^	
	*B*	(95% CI)	*B*	(95% CI)
Decision-making autonomy (Tertile)				
Highest (Reference)	—	—	—	—
Average	−46.8	(−92.8, −0.96)*	−30.0	(−75.2, 15.1)
Lowest	−87.9	(−132.6, −43.2)**	−68.8	(−114.4, −23.2)**

^1^Adjusted for maternal age, BMI, asset scores, maternal education, and husband's education.

*P value at <0.05, **P value at <0.01.

**Table 4:** Association of LBW with decision-making autonomy using binary logistic regression with crude and adjusted models; odds ratio (OR) and 95% confidence intervals (CI).

	Model with autonomy only		Full model	
	Crude OR	(CI)	Adjusted OR^1^	(CI)
Decision-making autonomy (Tertile)				
Highest	1	Reference	1	Reference
Average	1.30	(1.0, 1.6)*	1.24	(0.9, 1.6)
Lowest	1.46	(1.1, 1.8)**	1.40	(1.0, 1.8)*

^1^Adjusted for maternal age, asset scores, maternal education and husband education.

*P value at <0.05, **P value at <0.01.
